# Data-driven identification of complex disease phenotypes

**DOI:** 10.1098/rsif.2020.1040

**Published:** 2021-07-28

**Authors:** Markus J. Strauss, Thomas Niederkrotenthaler, Stefan Thurner, Alexandra Kautzky-Willer, Peter Klimek

**Affiliations:** ^1^ Complexity Science Hub Vienna, Josefstädter Straße 39, 1080 Wien, Austria; ^2^ Unit Suicide Research and Mental Health Promotion, Department of Social and Preventive Medicine, Center for Public Health, Medical University of Vienna, Kinderspitalgasse 15, 1090 Wien, Austria; ^3^ Section for Science of Complex Systems, CeMSIIS, Medical University of Vienna, Spitalgasse 23, 1090 Wien, Austria; ^4^ Department of Endocrinology and Metabolism, Internal Medicine III, Medical University of Vienna, Spitalgasse 23, 1090 Wien, Austria; ^5^ Santa Fe Institute, 1399 Hyde Park Road, Santa Fe, NM 85701, USA

**Keywords:** comorbidity, multimorbidity, electronic health records, disease network, obesity

## Abstract

Disease interaction in multimorbid patients is relevant to treatment and prognosis, yet poorly understood. In the present work, we combine approaches from network science, machine learning and computational phenotyping to assess interactions between two or more diseases in a transparent way across the full diagnostic spectrum. We demonstrate that health states of hospitalized patients can be better characterized by including higher-order features capturing interactions between *more* than two diseases. We identify a meaningful set of higher-order diagnosis features that account for synergistic disease interactions in a population-wide (*N* = 9 M) medical claims dataset. We construct a *generalized disease network* where (higher-order) diagnosis features are linked if they predict similar diagnoses across the whole diagnostic spectrum. The fact that specific diagnoses are generally represented multiple times in the network allows for the identification of putatively different disease phenotypes that may reflect different disease aetiologies. At the example of obesity, we demonstrate the purely data-driven detection of two complex phenotypes of obesity. As indicated by a matched comparison between patients having these phenotypes, we show that these phenotypes show specific characteristics of what has been controversially discussed in the medical literature as metabolically healthy and unhealthy obesity, respectively. The findings also suggest that metabolically healthy patients show some progression towards more unhealthy obesity over time, a finding that is consistent with longitudinal studies indicating a transient nature of metabolically healthy obesity. The disease network is available for exploration at https://disease.network/.

## 1. Introduction

Traditionally, medical research and practice follow a reductionist [1] specific-disease approach that often neglects that hospitalized patients usually suffer from a variety of diseases [2], a phenomenon called multimorbidity [3]. Interactions between multiple diseases may have severe consequences both for diagnosis and treatment and often form decisive features of the clinical picture of the patient [4]. For clinical practice, it is important to disentangle these complex disease relationships, which are far from fully understood [5].

One approach to better understand the relationships between diseases are comorbidity networks. Comorbidity networks formalize disease co-occurrences by representing diagnoses as nodes and linking diagnoses that tend to co-occur in patients [6–8]. In comorbidity networks, the health state of a multimorbid patient is characterized by multiple diagnoses that appear in network clusters (i.e. groups of nodes with more links within the group than to nodes outside of the group) of e.g. mental, metabolic or cardiovascular diseases [9,10]. So far, this strand of literature on comorbidity networks typically focused on pairwise co-occurrence patterns. This approach may miss complex interactions between diseases. The investigation of multimorbidity patterns has accordingly been defined as a priority in this line of research [8].

Besides the comorbidity network approach, recent years have brought increased activity in disease risk modelling using a plethora of data mining and machine learning approaches [11–15]. Machine learning is primarily dedicated to the prediction of individual diseases or groups of closely related diseases [13,15]. Different from network approaches it applies a broad spectrum of diagnostic information. These methods have yielded high predictive performance, but their transparence and interpretability are often limited [16,17]. Even with strategies such as *post hoc* interpretation [17], these approaches to the best of our knowledge cannot be used to address complex interactions across the whole disease spectrum which we pursue in the present study.

Previous work has described approaches for gathering phenotypic descriptions of patients and discovering correlations between diseases, see e.g. Roque *et al*. [18]. These studies typically use a variety of categories of medical records to analyse relationships between diseases [19]. To date, however, studies in the area do not allow to explicitly study interactions between more than two diseases, so-called higher-order interactions, and often use data from only one hospital [18]. In this study, we use the terms higher-order and complex interactions interchangeably. We further distinguish two types of interaction. In synergistic interactions, the combined effect of two or more diseases is higher than what would be expected from the individual diseases; in redundant interactions, the overall combined effect is reduced.

We aim to identify *disease phenotypes* from diagnosis data in population-wide electronic health records. For a specific *index disease*, there might be several disease phenotypes which differ regarding their comorbidity context, i.e. their location and neighbourhood in the disease network. A specific location in the network is related to a specific risk of acquiring further diseases (see below).

Obesity is controversially discussed in the medical literature to form different phenotypes. Using obesity as an example index disease, we evaluate if and to what extent the patients assigned to the two major obesity clusters on the generalized disease network differ in terms of co-morbidities and prognosis. For this purpose, we use a case–control design with cases and controls matched by age, sex and place of residence. We find that the main obesity clusters are largely consistent with what has been discussed in the medical literature as metabolically healthy and unhealthy obesity, respectively. Different from clinical studies in the topic area, which typically follow obese and non-obese patients over time, we show for the first time, that obesity clusters in ways that are somewhat consistent with the discussed clinical phenotypes.

Here, we integrate approaches from network science, machine learning and computational phenotyping to assess complex higher-order interactions between diseases. The proposed method is transparent and comprehends diseases across the full diagnostic spectrum in a way that leads to clinically meaningful results.

### 1.1. Definitions

The following terms are used throughout the paper with a specific meaning. To improve readability, we provide these definitions here:

— Disease: A condition that impairs normal functioning and is typically manifested by distinguishing symptoms and coded by a diagnosis.
— Diagnosis: A code in the International Classification of Diseases, typically encoding a disease, injury or symptoms.
— Diagnosis feature: One or more diagnoses with similar co-occurring diagnoses (i.e. comorbidities) and similar progression. Diagnosis features are of ‘higher order’ if they consist of more than a single diagnosis. In this work, they are the basis for capturing the higher-order interactions between diagnoses and the corresponding diseases. They allow a more fine-grained picture of the human disease network when compared with single diagnoses.
— (Optimal) feature set: A (meaningful) set of higher-order diagnosis features. The optimal (meaningful) feature set is characterized by maximizing the predictive performance of a cross-validated multinomial naive Bayes model through the tuning of few hyperparameters. The model inputs are the diagnosis features from the feature period as predictors, and the single diagnoses from the subsequent target period as predicted targets.
— Generalized disease network: A network where the nodes are diagnosis features representing one or more diagnoses and with weighted links representing the similarity of the model coefficient vectors associated with the linked nodes. Stronger links represents higher similarity of the disease features with respect to their predicted target diagnoses.
— (Putative) disease phenotype: A cluster of diagnosis features of a given index disease on the generalized disease network. A specific disease phenotype might indicate shared aetiological factors between the diseases reflected in the included diagnoses. Furthermore, a disease phenotype better reflects the original diseases than single diagnoses with respect to aetiological factors.
— Index disease: A specific diagnosis code of interest to explore comorbidities and identify disease phenotypes.

### 1.2. Data and design summary

A hospital population of 9 M patients was considered. The criteria for selection into the study were having no in- or outpatient hospitalization for a duration of 6 years, followed by at least one hospitalization during the next 3 years (the *feature period*), followed by at least one further hospitalization during the following 3 years (the *target period*). Half a million patients were selected into the study population.

The goal of this study was to identify *disease phenotypes* through the use of diagnosis information in electronic health records. The first step was to identify meaningful *diagnosis features*, i.e. features maximizing the predictive power, capturing higher-order interactions between diagnoses. A diagnosis feature is understood as a set of one or more (co-occurring) diagnoses. A *meaningful set of diagnosis features* was identified from the data using the following approach: (a) we mapped the patients' single diagnoses of the feature period to diagnosis features; (b) we used them to predict the patients’ single diagnoses—the (*prediction*) *targets*—of the target period, and, finally, (c) we selected a set of features that maximized the predictive performance with respect to the target diagnoses. Using this feature set, a final predictive model was fit to the whole dataset, referred to as *all-data model*. The *non-trivial* information contained in the correlation structure of the model coefficients was used to produce a comorbidity network where the nodes are sets of diagnoses (the *diagnosis features*) instead of single diagnoses. We call this the *generalized disease network*. There, disease phenotypes could be identified using clusters of diagnosis features. This was demonstrated at the example of the index disease obesity, where we propose to interpret clusters of obesity features as different disease phenotypes of obesity. Indeed, a matched comparison of patient cohorts corresponding to these obesity clusters showed that two of these clusters showed characteristics of metabolically healthy and unhealthy obesity, respectively.

## 2. Results

### 2.1. Identifying meaningful diagnosis features

**Figure 1.**
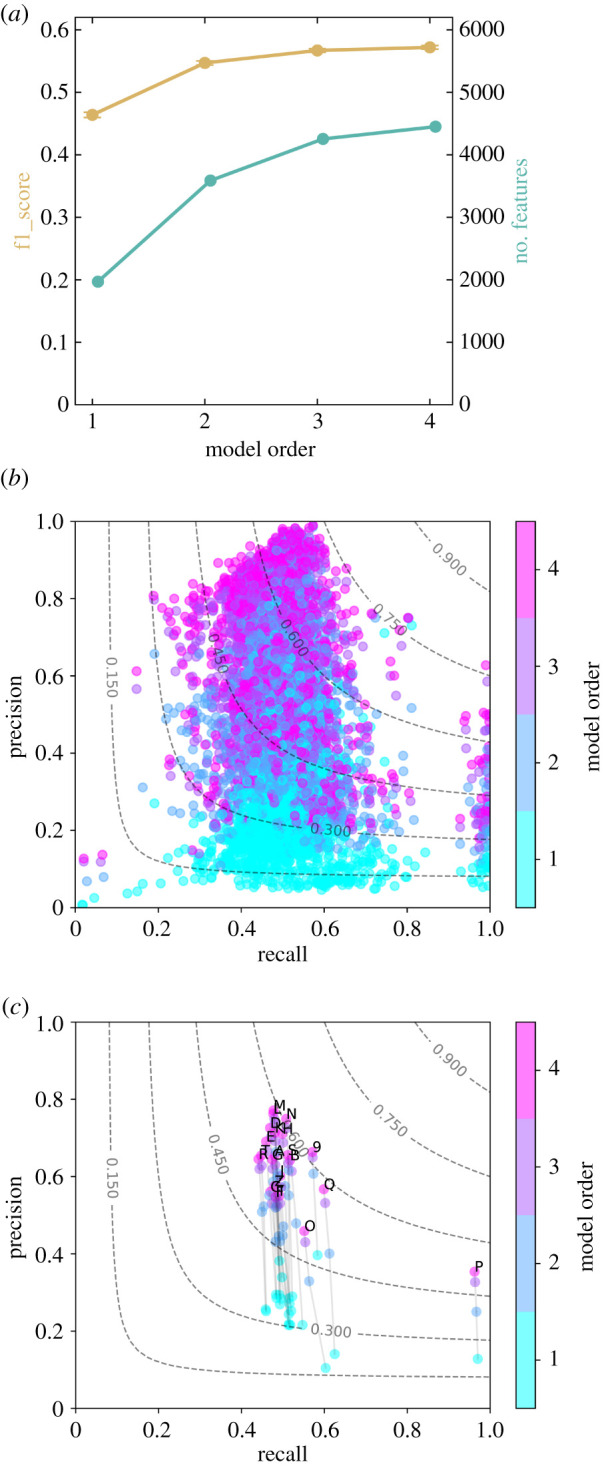
Higher-order diagnosis features improve disease predictions. This figure shows the in-sample metrics of the all-data model. Panel (*a*) shows the support-weighted average F1 score over the 1000 diagnosis targets with highest support (ochre line, error bars denote the standard error of the mean) and the size of the optimal feature set as a function of model order (turquoise). (The model order equals the maximally allowed number of diagnoses in one feature.) Both F1 and the number of features increase with increasing model order. Panels (*b*) and (*c*) show precision and recall of the 1540 naive Bayes classifiers for model orders one to four. Note that these are the target diagnoses that occur in at least 200 patients in all folds for all model orders. Each dot represents a single target diagnosis (*b*) or the average over ICD-10 groups (based on first character of respective ICD code) of target diagnoses (*c*). The dot colour encodes model order ranging from one (cyan) to four (magenta) and models of different order for the same diagnoses are connected by grey lines (*c*). The contour lines of constant F1 are dashed. (*b*) We find large heterogeneity in the quality of the individual naive Bayes classifiers. (*c*) Increases in model order lead to substantial increases in precision at similar levels of recall. We observe different classifier qualities for different groups of diagnoses.

Figure 1 demonstrates that the use of increasingly complex (i.e. higher order) features indeed improves the average predictive performance of a multinomial naive Bayes model; see §5. Figure 1*a* shows the model quality in terms of the total average F1 score as a function of the maximal number of diagnoses that may make up a feature, i.e. the *model order*. We computed models up to model order four. Figure 1*a* also shows that the number of features in the optimal set increases with model order. Figure 1*b* shows that precision and recall vary substantially across different diagnoses, almost over the entire range of possible values between zero and one. Individual F1 scores range between 0.04 and 0.78. This means that some diagnoses can be predicted very well from the feature information, others less. Among those diagnoses with the highest prediction accuracies, we find diagnosis codes (ICD-10) starting with M (diseases of the musculoskeletal system and connective tissue) and N (diseases of the genitourinary system), whereas the lowest scores are found for codes starting with O (pregnancy, childbirth and the puerperium) and C (malignant neoplasms).

In figure 1*c*, diagnoses are grouped by the first character of their respective ICD-10 code. Higher model orders clearly increase precision while keeping the recall approximately constant. Overall, the resulting increase in F1 is therefore mostly due to the increase in precision. For example, diagnoses starting with M (diseases of the musculoskeletal system and connective tissue), L (diseases of the skin and subcutaneous tissue) or N (diseases of the genitourinary system) show high precision on average, whereas diagnoses starting with P (conditions relating to the perinatal period), O (pregnancy, childbirth and the puerperium) or F (mental and behavioural disorders) have the lowest. We find a recall close to one for codes of group P while most other groups have recalls around 0.5.

The high recall of group P very likely is an artefact because newborns with P-diagnoses are preferentially selected into the *feature period* rather than the *target period*; see §5. A low number of new P-diagnoses in the target period together with the monotonicity assumption leads to high recall.

For an overview of all diagnoses and diagnosis groups with their respective scores, electronic supplementary material, Scores.xlsx.

### 2.2. Quantifying disease interactions

An immediate by-product of the computation of the model coefficients is the quantifiability of synergistic effects between diagnoses, presented in table 1. Synergistic and redundant effects are measured through their *lift*. Lift is computed from multiple models of orders one to four. It is the difference of a measured model coefficient and its expectation based on the coefficients of its respective lower-order models. For example, the lift of feature ‘E11 s E14 s E66s’ is computed from its coefficient of model order three and the coefficients of features ‘E11s to E14s’ from model order two and ‘E66s’ from model order one, respectively. Like the model coefficients, the lift is computed for specific feature-target combinations. The model coefficients, and thus lift, correspond to log-odds and are herein measured in decibans (*db*, see §5). A positive lift means an increase in log-odds compared to its expectation. Table 1 presents feature-target combinations that occur in at least 50 patients, thus reducing fluctuations of the model coefficients, and shows combinations with coefficients of at least 8 db, reflecting a relatively large effect size.

**Table 1.** Highest-ranking (by median lift) feature–target combinations. This table shows the highest-lift features and their corresponding target diagnoses. Included are diagnosis features comprising two or more diagnoses and where the feature–target combination occurs in at least 50 patients with a coefficient of at least 8 db. The median value of the lift is shown if there is more than one target fullfiling the selection criteria for a given feature. Note that some features with three or more diagnoses can be split into features with one or two diagnoses, respectively; these constituent features are separated by vertical bars. They provide the basis for the lift computation for the feature *f.*

feature, *f*	feature description	lift, *L*(*f, t)*	target(s), *t*	target description
E11s E14s | E66s	E11: type 2 diabetes mellitus, E14: unspecified diabetes mellitus, E66: overweight and obesity	3.0 db	I25s, I25p, I20p, I21p	I20: angina pectoris, I21: acute myocardial infarction, I25: chronic ischaemic heart disease
E66s | E79s	E66: overweight and obesity, E79: disorders of purine and pyrimidine metabolism	2.9 db	I25s, I25p	I25: chronic ischaemic heart disease
E11p E11s | E66s	E11: type 2 diabetes mellitus, E66: overweight and obesity	2.3 db	I25s	I25: chronic ischaemic heart disease
E66s | E11s	E11: type 2 diabetes mellitus, E66: overweight and obesity	1.9 db	I25p	I25: chronic ischaemic heart disease
I50p I50s | N18s	I50: heart failure, N18: chronic kidney disease (CKD)	1.9 db	E78s	E78: disorders of lipoprotein metabolism and other lipidaemias
E66s | I11s	E66: overweight and obesity, I11: hypertensive heart disease	1.9 db	I25s	I25: chronic ischaemic heart disease
E79s N18s | N39s	E79: disorders of purine and pyrimidine metabolism, N18: chronic kidney disease (CKD), N39: other disorders of urinary system	1.9 db	E11s, I25s	E11: type 2 diabetes mellitus, I25: chronic ischaemic heart disease
E79s | E14s	E14: unspecified diabetes mellitus, E79: disorders of purine and pyrimidine metabolism	1.5 db	I25s	I25: chronic ischaemic heart disease
G30s | F00s	G30: Alzheimer's disease, F00: dementia in Alzheimer's disease	1.3 db	F05p, S72p	F05: delirium due to known physiological condition, S72: fracture of femur
E79s | I11s	E79: disorders of purine and pyrimidine metabolism, I11: hypertensive heart disease	1.2 db	I25s	I25: chronic ischaemic heart disease
F17s | I21p	F17: nicotine dependence, I21: acute myocardial infarction	1.0 db	I20p	I20: angina pectoris

Note that findings in table 1 should be considered as exploratory only and do not include testing for statistical significance. Positive (negative) lifts indicate synergistic (redundant) interactions. We present the features with the largest synergistic effects across all feature–target combinations. Our model contains trivial predictions where a feature is used to predict a target already contained in that feature's diagnosis set. Note that the model incorporates a monotonicity assumption: once a patient has received a diagnosis, we assume the condition remains positive throughout. Disregarding those trivial associations, the top 10 features of at least two diagnoses with highest lifts, grouped by feature and averaged over all targets to which the respective feature contributes, are shown in table 1. In general, many of the synergistic effects contain specific components of the metabolic syndrome and cardiovascular target variables—associations that are well established in the literature. For instance, disorders of purine and pyrimidine metabolism (E79), specifically hyperuricaemia, have frequently been described as a marker of metabolic syndrome [20] and are part of several synergistic effects. For example, the combination of E79 with N18 (chronic kidney disease), if also combined with N39 (other disorders of the urinary system) is associated with an average lift of 1.9 db for the targets E11 (diabetes mellitus) and I25 (chronic ischaemic heart disease). This corresponds to 1.5-fold increased odds of positive target diagnoses E11 and I25, when N39 is additionally diagnosed together with E79 and N18 in the feature period. This combination of diagnoses constitutes the most complex example in table 1 and a full explanation is out of scope of this work. Regarding some of the included features, for example, hyperuricaemia (part of E79) has been shown to contribute to the progression of kidney disease in diabetes [21], with serum uric acid being either merely a marker of kidney damage or having a causal pathogenic role [22]. Furthermore, hyperuricaemia is associated with both chronic kidney disease and chronic ischaemic heart disease [22]. Accumulating evidence points to a possible aetiologic role of increased uric acid in the pathogenesis of cardiovascular disease [8]. A recent meta-analysis further found that every 1 mg dl^−1^ increase in serum uric acid was related to a 12 % increase in cardiovascular mortality in chronic kidney disease patients [23].

Several other noteworthy synergistic effects are present, e.g. a combination of N18 (chronic kidney disease) and I50 (heart failure) with regard to E78 (disorders of lipoprotein metabolism; 1.9 db, i.e. change in odds of 1.5). This is consistent with literature that shows a link between chronic kidney disease with heart failure, and with hyperlipidaemia, respectively [24,25]. Pathophysiological features indicate that heart failure can cause a reduction in cardiac output and decrease in renal perfusion which are primary drivers of renal dysfunction in heart failure. Among the various confounding factors for renal dysfunction in heart failure, dyslipidaemia has received increasing attention, and its role has not been fully understood [25].

Other displayed synergistic effects include a combination of two diagnoses for Alzheimer's disease (F00, G30) which increases the odds of fracture of femur (S72) and delirium (F05) (1.3 db, i.e. change in odds of 1.3). Accordingly, Alzheimer's disease has been shown to be an important risk factor for serious falls, including pelvic and femur fracture [26–28]. Furthermore, a review found that superimposed delirium among populations with dementia was highly prevalent [29]. Delirium and Alzheimer's disease are frequent causes of cognitive impairment among older adults and share a complex relationship in that delirium and Alzheimer's disease can occur independently, concurrently, and interactively, for example, delirium can alter the cause of an underlying Alzheimer's disease. Models for a shared pathophysiology of delirium and Alzheimer's disease have recently been proposed, including common baseline risk biomarkers and outcome biomarkers [30].

There is a synergy between hyperuricaemia (E79) and hypertensive heart disease (I11); which are both risk factors for chronic ischaemic heart disease (I25; 1.3 db). While hypertension is a well-established risk factor for chronic ischaemic heart disease, also hyperuricaemia has been established as an independent risk factor [31,32]. To the best of our knowledge, the combined risk of these risk factors has not yet been evaluated.

A further synergistic effect is found between smoking (F17) and acute myocardial infarction (AMI, I21) with regard to angina pectoris (I20, 1.0 db, i.e. change in odds of 1.3). Studies show accordingly that smoking after AMI is associated with a considerably increased risk of more angina [33].

### 2.3. The generalized disease network

**Figure 2.**
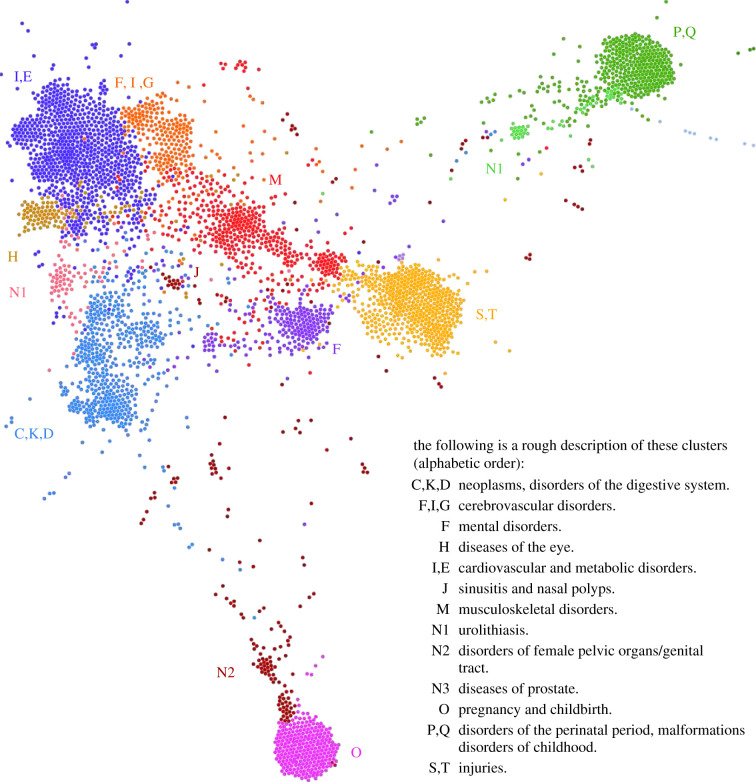
Generalized disease network. Nodes correspond to diagnosis features. The presence of links and their strength determine the computations of both the network layout (Gephi/ForceAtlas2) as well the clustering (Louvain). Nodes tend to be in closer proximity and/or belong to the same cluster if they have higher connectivity. Links are omitted for readability. A distinct property of this disease network is that single diagnoses generally occur in multiple features. Colour coded are the different clusters in the network. A cluster generally includes diagnoses from different ICD chapters. Upper-case letters mark the main locations of diagnoses in specific ICD-10 chapters. The network can be explored interactively at https://disease.network/.

**Figure 3.**
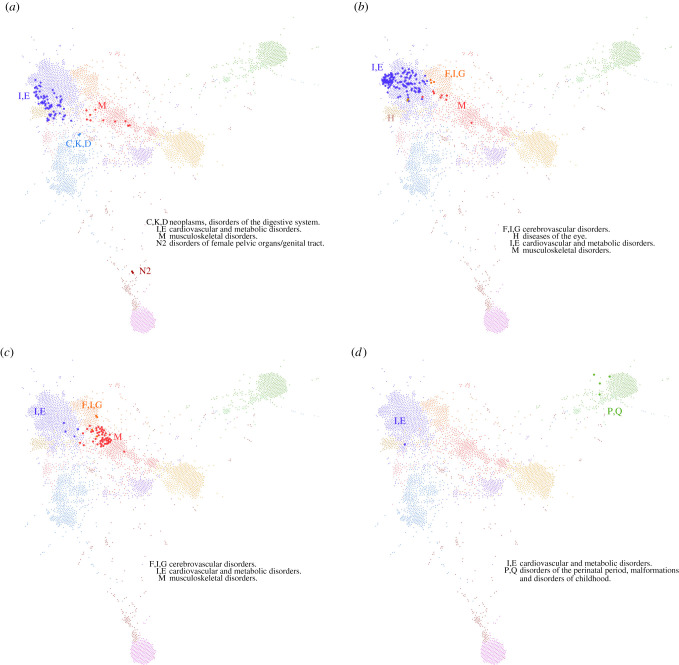
Selected diagnoses on the generalized disease network. Here, we show how specific selected diagnoses are distributed across the network. (*a*) E66—Obesity, takes part in four clusters. Of all E66-features, 82% are part of the cluster of mainly cardiovascular diseases and metabolic disorders (top-left, ‘I,E’ in dark blue); 14% are part of the cluster of mainly musculoskeletal disorders (‘M’ in red). These two groups of features formed the basis of our matched-cohort analysis. The blue obesity cluster was identified with the MUHO phenotype, the red cluster with MHO. Additionally, there are two more groups, each with two features: one in the cluster of neoplasms and disorders of the digestive system (‘C,K,D’ in light blue) and the other in the cluster of disorders of female pelvic organs and genital tract (bottom, ‘N2’ in brown). (*b*) I25—Chronic ischaemic heart disease, is primarily spread across four clusters, with most of its features being part of the cluster of mainly cardiovascular diseases and metabolic disorders (‘I,E’ in dark blue). Also, the clusters of mainly nervous diseases (‘F,I,G’ in orange), musculoskeletal diseases (‘M’ in red) and diseases of the eye (‘H’ in ochre) host I25-features. (*c*) M81—Osteoporosis without current pathological fracture, is arranged in the musculoskeletal cluster (‘M’ in red) interspersing into the neighbouring clusters of mainly nervous diseases (specifically Parkinson's disease, dementia and depression; ‘F,I,G’ in orange) and cardiovascular/metabolic disorders (‘I,E’ in dark blue). (*d*) J45—Asthma, is contained in two clusters. First, in the cluster of disorders of the perinatal period, malformations and disorders of childhood (top right, ‘P,Q’ in green) and of cardiovascular/metabolic disorders (top left, ‘I,E’ in dark blue). In the ‘P,Q’ group, direct comorbidities of Asthma are J44 (COPD) and J18 (Pneumonia), in the ‘I,E’ group the direct comorbidity of the single feature is J44 (COPD).

The diagnosis features which capture higher-order interaction effects are used to inform the construction of the generalized disease network. See figure 2 for an overview. There, nodes correspond to diagnosis features and links between features (omitted in the figure) indicate that these features predict similar target diagnoses; see §5. There are well discernible clusters of features roughly corresponding to the chapters of the ICD-10 classification and summarized in figure 2. Specific examples for the distribution of obesity (E66), chronic ischaemic heart disease (I25), osteoporosis (M81) and asthma (J45) are presented in figure 3.

### 2.4. Detecting obesity phenotypes

To detect disease phenotypes, we perform a network community detection, i.e. clustering. Clusters are identified such that nodes (diagnosis features) are more strongly connected within their respective cluster than with nodes outside their clusters. We expect that different disease phenotypes present themselves in different comorbidity contexts and different prognoses. We, therefore, expect them to be located in different clusters of the network. This means that for a given index disease we expect to find distinct disease phenotypes in different clusters (colours) of the generalized disease network.

We find the index disease obesity (E66) distributed across four different clusters in the network. We use the two largest clusters to construct a matched case–control design, see §5. We compare them with non-obese controls. Individuals in one of the clusters show characteristics of metabolically unhealthy obesity, whereas the other cluster is metabolically more healthy [34–37] (figure 3*a*). In particular, patients in the cluster of metabolically unhealthy obesity (MUHO candidates), show a considerably higher prevalence of diabetes, hyperlipidaemia, when compared with both, patients in the cluster of metabolically healthy obesity (MHO candidates) and non-obese controls. Hypertension, ischaemic heart diseases and other forms of heart disease already have higher prevalences among MHO candidates, compared to controls. With regard to these diseases, MHO candidates have an intermediate position between control and MUHO candidates. MHO candidates, however, are similar to controls in terms of diabetes and hyperlipidaemia prevalence as well as mental and behavioural disorders due to psychoactive substance use (including nicotine dependence), which are all increased in MUHO candidates. Table 2 displays metabolic and selected further diagnoses with significant differences in prevalence for obesity phenotypes and controls. Note that, based on approx. 200 blocks in the dataset, significant differences as indicated with ** or *** in table 2 remain significant after adjustment for multiple testing. The phenotypes do not differ in terms of number of diagnoses or days in hospital, with both groups having more diagnoses and longer stays when compared with controls.

**Table 2.** Comparison of putative metabolically healthy obesity (MHO) and metabolically unhealthy obesity (MUHO) cohorts with non-obese controls. The effect size (ES) is measured in decibans (db) computed as ES_*i*,*j*_: *=* 10(log_10_ *n_j_−* log_10_ *n_i_*) db, where *i, j* are the group indices with 0 = control, 1 = MHO cand., 2 = MUHO cand., and *n_k_* is the relative frequency of patients in group *k* with at least one diagnosis of the corresponding diagnosis block (shown as percentages). A value of ES_*i,j*_ > 5 db can be considered substantial evidence, ES_*i,j*_ > 10 db as strong, ES_*i,j*_ > 15 db as very strong and ES_*i,j*_ > 20 db as decisive evidence.^†^ Asterisks indicate the *p*-value of the effect size (G-test): *p *<* 10^−2^; **p *<* 10^−4^ and ***p *<* 10^−6^.

	**sex**	**0: controls**	**ES_0_**_,_**_1_** (db)	**1: MHO cand.**	**ES_1_**_,_**_2_** (db)	**2: MUHO cand.**
before matching						
number of patients	f	252 708		710		7789
	m	208 990		221		7003
birth year, mean (SEM)	f	1959.77(5)		1942.4(6)		1946.22(20)
	m	1961.89(5)		1945.2(10)		1949.35(19)
after matching						
number of patients	f	6840		684		2052
	m	2060		206		618
birth year, mean (SEM)	f	1941.90(17)		1941.9(5)		1941.91(30)
	m	1945.24(29)		1945.3(9)		1945.3(5)
no. of hospitalization days per patient (during feature and target periods)	f	35.7(5)		54.0(23)		52.1(12)
	m	33.8(9)		45.9(33)		50.0(21)
primary diagnoses per patient during feature period	—	1.73		2.37		2.45
secondary diagnoses per patient (T1)	—	2.77		6.60		6.93
in-hospital mortality (9 year follow-up)	—	12%(1093)		14%(127)		15%(412)
prevalences of selected diagnosis blocks (during the feature period)						
diabetes mellitus (E10–E14)	f	8.3%(567)	*−*1.0	6.6%(45)	6.8***	32%(650)
	m	10%(206)	*−*0.1	9.7%(20)	5.3***	33%(202)
metabolic disorders (E70–E90)	f	14%(934)	*−*0.1	13%(92)	5.1***	44%(897)
	m	16%(326)	0.2	17%(34)	5.2***	55%(340)
mental and behavioural disorders due to psychoactive substance use (F10–F19)	f	2.2%(153)	*−*1.4	1.6%(11)	5.6***	5.9%(121)
	m	6.9%(143)	*−*2.5	3.9%(8)	5.4**	14%(84)
hypertensive diseases (I10–I15)	f	27%(1818)	3.5***	60%(411)	0.7*	71%(1454)
	m	27%(547)	3.9***	65%(133)	0.6	74%(455)
ischaemic heart diseases (I20–I25)	f	7.4%(509)	2.5**	13%(90)	1.8*	20%(413)
	m	12%(244)	2.7*	22%(45)	1.8	33%(202)
other forms of heart disease (I30–I52)	f	10%(697)	2.8***	19%(132)	0.8	23%(473)
	m	11%(224)	4.1***	28%(57)	0.2	29%(179)
incidences of selected diagnosis blocks (during the target period)						
diabetes mellitus (E10–E14)	f	7.5%(513)	0.5	8.3%(57)	3.4***	18%(374)
	m	9.4%(193)	1.3	13%(26)	1.9	20%(122)
metabolic disorders (E70–E90)	f	13%(900)	0.2	14%(95)	0.5	16%(322)
	m	13%(267)	1.2	17%(35)	*−*0.4	16%(96)
mental and behavioural disorders due to psychoactive substance use (F10-F19)	f	1.9%(133)	0.5	2.2%(15)	*−*0.5	1.9%(40)
hypertensive diseases (I10-I15)	f	20%(1387)	*−*0.9	17%(113)	0.4	18%(373)
	m	20%(403)	*−*0.9	16%(33)	0.8	19%(118)
ischaemic heart diseases (I20–I25)	f	8.6%(591)	*−*0.3	8%(55)	1.4	11%(230)
	m	13%(259)	*−*0.9	10%(21)	1.9	16%(97)
other forms of heart disease (I30–I52)	f	12%(838)	1.7**	18%(125)	0.1	19%(381)
	m	13%(275)	2.2*	22%(46)	*−*0.2	21%(132)

^†^See [38], p. 423).

Regarding the incidence of new metabolic and cardiovascular diseases during follow-up, again MHO patients have a position in-between MUHO and control patients. Metabolic diseases, most importantly diabetes, are more frequently newly diagnosed among patients that already were in the MUHO cluster.

In-hospital mortality over a follow-up period of 9 years did not significantly differ between MHO and MUHO patients, and mortality of MHO patients was again in-between that of non-obese controls and MUHO patients.

It is straight-forward but beyond the scope of this work to extend the above computational approach to other index diseases than obesity. In particular, we show in figure 3*b*–*d* that also (b) chronic ischaemic heart disease, (c) osteoporosis and (d) asthma might show distinct disease phenotypes in multiple clusters. The phenotypes of more than 100 different index diseases can be explored online at https://disease.network/.

## 3. Discussion

The goal of this study was to develop a method to automatically identify *disease phenotypes* through the use of diagnosis information in electronic health records. In the first step, we identified *diagnosis features*, i.e. meaningful sets of diagnoses, to capture higher-order interactions of co-occurring diagnoses. We showed that taking higher-order interactions into account improved the performance of predicting disease progression.

The second step was to display these higher-order interactions by constructing a network of diagnosis features with the best predictive performance and tessellate it into clusters. As diagnosis codes are generally part of multiple diagnosis features, the way how specific diagnoses are distributed across the network in clusters might indicate different disease phenotypes.

There is, to the best of our knowledge, no previous literature which has investigated the impact of higher-order interactions between diagnosis codes across the full diagnostic spectrum. Previous studies for network construction [7] have only looked at pairwise co-occurrence patterns of diagnoses. In those networks, each diagnosis is only represented once. This single representation cannot differentiate between *multiple* phenotypes of the respective index disease. Complex diseases, however, have in common that they (a) cannot be captured by one single specific diagnosis (e.g. metabolic syndrome) and (b) that a specific diagnosis can present itself as part of multiple disease phenotypes (e.g. hypertension). The approach developed here allows us for the first time to investigate multiple roles of specific diagnoses in disease phenotypes, thereby filling a methodological chasm in the current literature on multimorbidity [8].

At the examples of obesity (E66), chronic ischaemic heart disease (I25), osteoporosis (M81) and asthma (J45), we show that many complex diseases are indeed distributed across multiple clusters on the disease network (figure 3). For example, osteoporosis (figure 3*c*) occurs in clusters of cardiovascular and metabolic diseases and also in the neurodegenerative disease cluster, suggesting different (disease) phenotypes of osteoporosis. In accordance, cardiovascular [39,40], but also depression [41] and Parkinson's disease [42] have been identified as risk factors for osteoporosis. Shared pathways of both neurodegeneration with osteoporosis [43] and cardiovascular disease with osteoporosis [39] have been discussed, supporting the identified disease phenotypes. The latter has been hypothesized with common features between bone mineralization and atherosclerotic calcification [39].

Similarly, asthma is part of two clusters (figure 3*d*), suggesting the existence of two distinct asthma phenotypes. The first cluster is the one of mainly cardiovascular diseases (I,E in dark blue). Asthma has been identified as a risk factor for cardiovascular diseases [44]. In this cluster in the generalized disease network, asthma is primarily connected to chronic obstructive pulmonary disease which further connects to a phletora of cardiovascular diseases. Asthma is a chronic inflammatory disease, with inflammatory processes being key in the pathophysiology of atherosclerotic diseases [44]. The second cluster is closely related to childhood diseases (P,Q in green), whereas a specific asthma phenotype, particularly as an early-onset allergic type of asthma, has been discussed for children [45]. Accordingly, there is a link in the generalized disease network between the features J45p (asthma) and J30s (vasomotor and allergic rhinitis).

### 3.1. Metabolically healthy versus unhealthy obesity phenotypes

Specifically for obesity (E66), we used the network to investigate obesity-related phenotypes. Obesity is a major public health problem associated with increased morbidity and mortality [46]. It represents a remarkably heterogeneous condition with different obesity-related comorbidities, impairment of functional status and varying cardiometabolic outcomes [47]. The concept of metabolically healthy obesity (MHO), in comparison to the metabolically unhealthy obesity (MUHO) phenotype is under debate and its relevance to clinical practice still unclear [46,47]. We could show that obesity features were indeed distributed across multiple clusters on the disease network. Depending on their cluster membership, patients showed some characteristic specific features—in terms of present and future comorbidity—of *metabolically healthy* and *unhealthy* obesity, respectively. MUHO represented the largest cluster with more than two thirds of the obese patients, corresponding very well to the general picture of this phenotype including metabolic comorbidities like dysglycaemia, dyslipidaemia, hypertension and/or hyperuricaemia and high cardiovascular risk. The second representative cluster (≈ 15 %) comprised obese patients with musculoskeletal problems and degenerative changes who feature hypertension but were otherwise metabolically healthy (MHO), again in accordance with epidemiological evidence [47,48]. Interestingly, before matching of the cohorts, we found female preponderance in the MHO cluster in accordance with other studies [49]. This may be ascribed to differences in sex hormones, body fat distribution, adipokines, immunological parameters, the microbiome and better insulin sensitivity of women compared to men [49]. Additionally, small clusters represented obese patients with gastrointestinal disorders and cancers or liver disease (including fatty liver, another candidate of the metabolic syndrome) or patients with reproductive or urogenital problems in our analysis, all well-known comorbidities based on obesity-related hormonal imbalance, inflammation and insulin resistance [50].

The identified diagnosis features contribute to the currently ongoing discussions regarding phenotype definitions of MHO [34–37,51]. In particular, we showed for the first time that diagnosis features of obesity are part of different clusters on a comorbidity network, and that these assignments are associated with strong differences in metabolic health, amplifying epidemiological evidence. A phenotype with low prevalence of diabetes and hyperlipidaemia seems to be distinct from a phenotype with high prevalence of hypertension, hyperlipidaemia and diabetes, which is linked to an increased risk of developing new metabolic and cardiovascular risk factors.

Furthermore, not only the prevalence of metabolic disorders was significantly higher in MUHO versus MHO but also that of mental disorders. It can be speculated that shared psychosocial factors are underlying pathophysiological mechanisms starting a vicious circle between unhealthy lifestyle, metabolic and psychological disturbances, which again are linked and mediated by obesity [49]. During follow-up, MUHO also had the highest incidence of diabetes which doubled in comparison to that of the MHO group. On the other hand, MHO was characterized by hypertension, highlighting that increased BMI is one of the most prominent causes of heart failure and ischaemic heart disease [52,53]. This might explain why both clusters showed a comparable incidence of ischaemic heart disease at follow-up. Also, mortality was increased in both clusters at follow-up without significant differences between the MHO and MUHO groups. This finding corroborates previous findings from cohort studies and surveys questioning the value of MHO for the determination of the overall prognosis of patients [54,55].

In total, the patterns identified give a mixed picture with regard to the discussions of a *benign* form of MHO, that is not at increased risk of negative cardiovascular prognosis and mortality [36,37,56]. Consistent with earlier research, an evaluation of the incidence of new metabolic and cardiovascular diseases during follow-up corroborates the picture that MHO patients have a position in-between MUHO and non-obese control patients, indicating a transient state [36]. The MHO disease phenotype identified is thus consistent with a more benign obesity phenotype, showing a slower progression to a more unhealthy obesity over time than the MUHO disease phenotype. Nevertheless, the MHO disease phenotype cannot be considered completely benign in terms of prognosis. This is corroborated by a meta-analysis of 22 prospective studies over a follow-up of 3.6–30 years, which did not identify any combinations of obesity and components of metabolic syndrome that were not at increased risk of cardiovascular events and mortality when compared with non-obese patients. The risk for MHO particularly increased for studies with longer-follow-up [36] suggesting that the majority of MHO patients converge towards the MUHO phenotype over time. Thus it is necessary to raise public awareness and to initiate lifestyle intervention in all MHO patients.

This elaborated example on obesity (E66) demonstrates how to use the developed method to obtain meaningful novel information about complex diseases. Different from clinical studies in the topic area, which typically follow obese and non-obese patients over time, we show for the first time, that the disease phenotypes of obesity identified in this purely data-driven approach are somewhat consistent with the discussed clinical phenotypes of obesity.

The generalized disease network is made available for public exploration at https://disease.network/.

### 3.2. Strengths and limitations

The two unique characteristics of our approach are (i) the computational identification of higher-order diagnosis features that (ii) simultaneously predict more than a thousand diagnoses, rather than focusing on one or a couple of diseases [15,57–59]. The present approach allows to describe the overall health state of inpatients across the boundaries of diseases and disease groups, providing a more holistic picture of their health-state. This facilitates the identification of phenotypes across diagnostic boundaries.

The following strategies were applied to improve validity: First, we performed fivefold cross-validation in the search for diagnosis features. This leads to meaningful diagnosis features. Second, the naive Bayes classifier is not prune to overfitting. We find comparable levels of model performance in the training and the test data, which suggests that overfitting is not an issue in our approach.

A study limitation is that only hospital diagnoses were available. Other clinical information such as prescriptions or performed medical procedures were not available. This work pertains exclusively to pattern detection in large datasets of diagnosis data which has been defined as a research priority [8]. The present study design was exploratory and appropriate to identify associations between diagnoses but not to infer causal relationships. Furthermore, cross-validated results are reported but cross-validation does not provide proper metrics for evaluation of prediction models. In the light of the study aim to identify disease phenotypes based on the contextualization of co-occurring diseases rather than predicting new diseases, this should, however, not pose a problem to the present analysis.

Because of the monotonicity assumption used and due to the discrepancies of disease onset and the timing of diagnosis which vary widely between diseases, it is not possible to make accurate timely predictions of new diagnoses in the target period based on the diagnosis features in the feature period. Based on the findings from other network analyses and the current patterns which suggest that diagnosis features are often related to diagnoses that represent later stages of the same or similar diseases it is however likely that many of the identified correlations reflect disease progression. We also show this explicitly for the index diagnosis of obesity, where metabolically health obesity approaches unhealthy obesity over the observation period. We found that without applying monotonicity, the target data were too sparse for the naive Bayes classifier to successfully capture higher-order associations. Also, we have been interested primarily in chronic diseases where this assumption is more valid.

**Figure 6.**
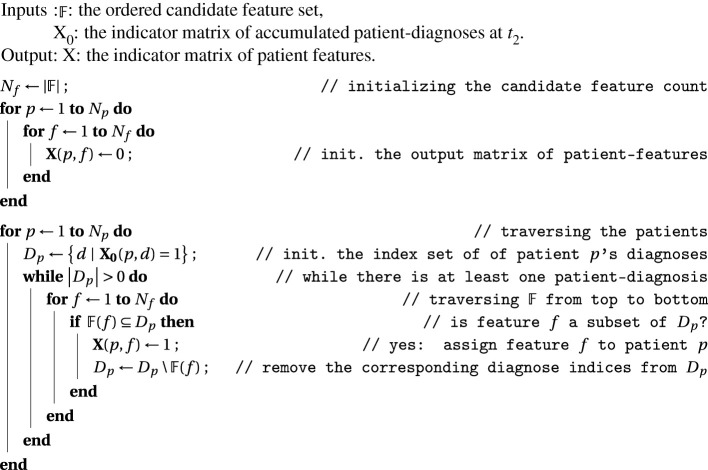
Algorithm to map patient-diagnoses to patient-features. Algorithm to map patient-diagnoses to patient-features. This algorithm corresponds to activity map_feature in electronic supplementary material, figures S5 (showing the usage context) and S6c (showing the algorithm in the form of an activity diagram).

Regarding computational cost, there are noteworthy methodological aspects which suggest that the computational cost is relatively moderate. In particular, the main point of our analysis method is that we identify statistical higher-order correlations in the data by applying (a) very efficient frequent itemset mining [60], (b) the generative classifier we use (naive Bayes) can be computed efficiently [61] and (c) the hyperparameter search applies the Bayesian optimization (BO) technique which is especially suitable for optimizing few variables efficiently with relatively few objective function evaluations; see [62] and references therein. The bottlenecks of the analysis are (a) the algorithm of mapping patient-diagnoses to patient-features, figure 6, and (*b*) the computation of the generalized disease network from the final (optimal) model coefficient matrix. Furthermore, a disadvantage of frequent itemset mining is that it can only detect a subset of possible higher-order associations, particularly the subset of positive associations. For higher-order associations, the positive-only associations mean a considerable saving in terms of computational cost, and a restriction to associations that are putatively most relevant in a sparse dataset.

## 4. Conclusion

In conclusion, based on a multinomial naive Bayes model, we demonstrate that health states of inpatients can be better characterized by the inclusion of higher-order features of multiple diseases. Diseases show a multitude of complex interaction effects among each other that generally impact a patient's disease progression in a non-additive way. The method developed allows to identify distinct disease phenotypes made up of clusters of diagnosis features which might correspond to different disease aetiologies that cannot be captured by single diagnoses and their interactions. This enables us to discover and analyse novel disease phenotypes based on different comorbidity contexts of diagnoses. The resulting differentiation has strong implications for a better understanding not only of disease aetiology but also for the meaning of a specific given diagnosis in terms of treatment and prognosis.

## 5. Material and methods

**Figure 4.**
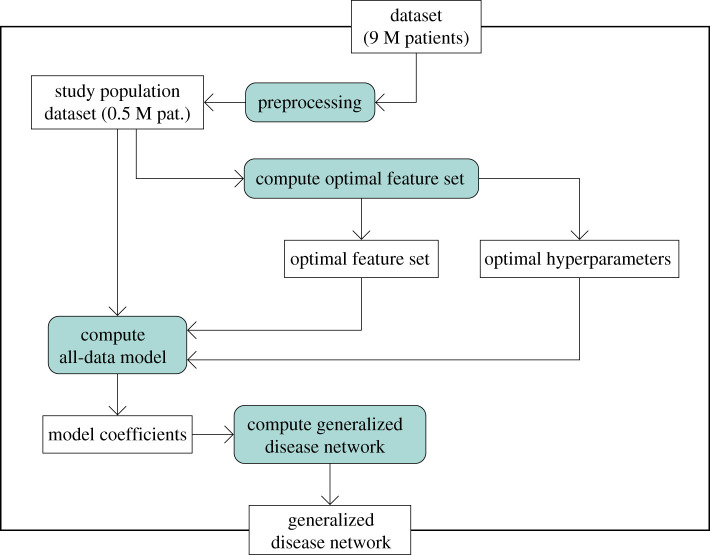
Overview of the modelling process. Rectangles denote input and output data for the processing steps which themselves are represented by rectangles with rounded borders and coloured background. The arrows indicate processing flow and data use.

First, we describe the main modelling process from the input dataset to the final generalized disease network, see figure 4 for an overview. Second, the matched cohort comparison of metabolically and unhealthy obese patients is described.

### 5.1. Dataset

A pseudonymized administrative dataset containing all in- and outpatient stays in privately and publicly funded hospitals in Austria over a time period of 18 years was used, covering patients with exit dates in 1 January 1997–31 December 2014. The dataset consisted of 45 M recorded stays of 9 M patients. Each stay record included a patient pseudonym, entry and exit dates, one primary diagnosis, zero or more secondary diagnoses, home region (34 categories), sex (two categories) and age group at the time of stay (5-year bins, 19 categories). The diagnoses were encoded as three character categories from the Austrian adoption [63] of the World Health Organization's international statistical classification of diseases and related health problems, 10th revision [64] (ICD-10). A transfer from one department to another resulted in a new stay record. In-hospital deaths were recorded together with a code for the declared reason of death.

### 5.2. Study population/preprocessing

**Figure 5.**
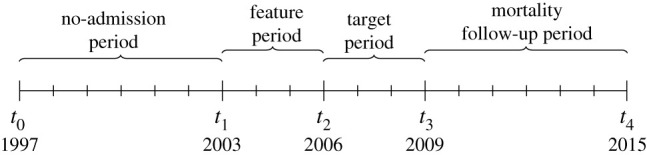
Timeline demonstrating the various time periods used in this study. The year dates represent midnight at 1st January of the respective year. Important points in time are named *t*_0_, … , *t*_4_.

The whole time period of 18 years was split into four parts for the purposes of predictive modelling and matched cohort analysis: the 6-year *no-admission period* from *t*_0_: = 1 January 1997 00:00 to *t*_1_ : =  1 January 2003 00:00, the 3-year *feature period* from *t*_1_ to *t*_2_ : =  1 January 2006 00:00, the 3-year target *period* from *t*_2_ to *t*_3_ : =  1 January 2009 00:00 and the 6-year *mortality follow-up period* from *t*_3_ to *t*_4_ : =  1 January 2015 00:00 (figure 5).

The criteria for selecting patients into the study were: (i) no recorded stay within the no-admission period, (ii) at least one recorded stay during the feature period, and (iii) at least one further recorded stay during the target period. This resulted in *N*_P _ =  478 575 patients who have been selected into the study. By the virtue of these criteria, we aim to capture patients that have no serious conditions (i.e. requiring hospitalization) during the last 6 years before entering the study, and are all alive at least until *t*_2_.

### 5.3. Identification of (meaningful) diagnosis features

In identifying the phenotypes of complex diseases in diagnosis records, we follow a two-step approach. The first step is to qualitatively (i.e. structurally) and quantitatively capture the higher-order statistical interactions between diseases. This step is described in this section. The second step is to construct a generalized disease network, described in §5.4.

We introduce *higher-order diagnosis features* as sets of diagnosis codes that serve as features in a predictive multitarget classification model. The features are computed from the patients' accumulated diagnosis codes that occurred up to t_2_ and are used to predict the accumulated diagnoses *(monotonicity assumption)* up to *t*_3_, i.e. the targets. The diagnosis codes are formed from the three-character ICD-10 codes, suffixed with the letter p or s if the code was used as a primary or secondary diagnosis, respectively.

Our aim is to identify a *meaningful* set of higher-order diagnosis features (henceforth called the feature set) in a computationally feasible way, by (I) applying a heuristic to construct the higher-order diagnosis features and (II) by selecting a feature set that maximizes the predictive performance (wrapper approach [65]). These steps are detailed as follows.

(I) **Heuristically constructing higher-order diagnosis features.** The patient diagnoses of the feature period are represented by the *N_P_*  ×  *N_D_* indicator matrix **X_0_**, with entries *X*_0_(*p*, *d*) for patient index *p* ∈ {1, … , *N_P_*} and diagnosis code index *d* ∈ {1, … , *N*_D_}, *N*_D_ being the total number of primary and secondary diagnosis codes. If patient *p* is diagnosed with *d* at least once in the feature period, we set *X*_0_(*p*, *d*)  =  1, otherwise *X*_0_(*p*, *d*)  =  0.
The data matrix **X_0_** is transformed into the feature matrix **X** by mapping the *N*_*D*_ diagnosis codes from the feature period to *N_F_* higher-order diagnosis features by (i) constructing a candidate feature set, (ii) greedily assigning features to patients, (iii) pruning features from the candidate feature set that occur in less than support_min _ =  30 patients, and repeating with (ii) until all features are supported at least by support_min_ patients.
(i) **Constructing a candidate feature set.** A feature candidate is a set of diagnoses that co-occur in patients. The candidate feature set is the ordered (see next paragraph) set of all candidate features. It is constructed heuristically as follows: We consider all combinations up to a specific number of combined diagnoses (the *model order*) and that occur at least in support_min_ patients. Furthermore, candidates are only included if their diagnoses occur more likely together than what would be expected from random chance. This is quantified using a generalized measure of mutual information, the ‘minimum information difference to prior’ (minIDP) measure which has to be above a threshold of minIDP_min_ (see electronic supplementary material, text 1). All of these steps are efficiently computed using a free open source frequent itemset mining software by Borgelt [66,67].
To obtain the ordered candidate feature set F, the feature candidates are sorted (i) from high to low cardinality, i.e. the number of diagnoses in a given feature, (ii) within same cardinality, from high to low minIDP and, (iii) within same (i) and (ii) from high to low support.
(ii) **Greedily assigning features to patients.** Patient-diagnoses are mapped to patient-features by applying a greedy heuristic whose algorithm is shown in figure 6.
(iii) **Pruning the candidate feature set and repeating.** After running the respective algorithm (figure 6), some (candidate) features may be weakly supported, i.e. their support drops below support_min_. Following the ordering of F, the first of those features is then removed and the algorithm is repeated using the newly pruned F, until the support of all assigned features is greater or equal support_min_. Nota bene, we remove only the first (versus all) of the weakly supported features because some of the features further down in F may become supported again simply by remapping the patients of the first weakly supported feature to other features. We will refer to the final ordered set of the well-supported higher-order features as final feature set. The patient-features corresponding to the final feature set are numerically represented as *N_P_* ×  *N_F_* matrix **X** with entries *X*(*p*, *f*)  =  1 if feature f has been assigned to patient *p*, and *X*(*p*, *f*)  =  0 otherwise. See electronic supplementary material, text 2 for more information.
(II) **Selecting the optimal feature set using the wrapper approach.** The selection of the optimal feature set using the wrapper approach consists of these components: (i) a predictive multi-target classification model that allows to score a given feature set, (ii) and a routine to select the tunable hyperparameters such that the score is maximized.
(i) **The multi-target classification model.** Based on a patient's features (computed from the accumulated diagnoses up to *t*_2_), we seek to predict his or her diagnoses obtained up to *t*_3_ (the prediction targets). The targets are represented by the *N_P_* ×  *N_D_* data matrix **Y**. If patient p was diagnosed at least once with target diagnosis *t* up to t_3_, we set *Y*(*p*, *t*)  =  1, otherwise *Y*(*p*, *t*)  =  0. As the target variables are binary, we require the choice of a classifier to be used in a multi-target predictor. We chose to use the multinomial naive Bayes classifier (MNB). Although MNB is often used for count data (e.g. word counts in natural language processing), it can also be used successfully for binary target variables.
For each feature *f* and target *t* and model order *m*, we obtain a model coefficient
  $$C^{\left(m\right)}(\;f,t)\;:=10\;\log_{10}\left(\frac{\mathrm{Pr}(X(\;p,f)=1|Y(\;p,t)=1)}{\mathrm{Pr}(X(\;p,f)=1|Y(\;p,t)=0)}\right)\;\mathrm{db},$$
  measured in decibans (db). The probabilities come from the model of order *m*. The superscript model order *m* is omitted where clear from context. C(*f*, *t*) is the estimated weight of evidence in favour of the presence of target diagnosis t in a patient provided by the presence of feature *f*. With other words, *C*(*f*, *t*) equals the change in the modelled log-odds [68]—of target diagnosis *t* present versus absent—due to the presence of feature f in a patient. A coefficient *C*(*f*, *t*) > 5 db (corresponding to a change in odds of ≈ 3 : 1) can be considered substantial evidence concerning *t* provided by *f*, *C*(*f*, *t*) > 10 db (10  : 1) as strong, *C*(*f*, *t*) > 15 db (≈ 30 : 1) as very strong and *C*(*f*, *t*) > 20 db (100 : 1) as decisive evidence. This scheme follows Jeffreys [38, p. 423].
The linear decision function of the classifier is
  $$\Delta (\;p,t)=\underset{\;f\;|X(\;p,f)=1}{\sum }C(\;f,t)+\pi (t),$$
  where *π*(*t*) are the prior log-odds,
  $$\pi (t)\;:=10\;\log_{10}\left(\frac{\mathrm{Pr}(Y(\;p,t)=1)}{\mathrm{Pr}(Y(\;p,t)=0)}\right)\mathrm{db}.$$
The target diagnosis *t* is classified *present* for patient *p* whenever Δ(*p*, *t*)  >  0, otherwise is classified *absent*. For target *t*, we evaluate prediction quality using precision (i.e. the probability that a predicted diagnosis actually occurred; the type 1 error rate), recall (i.e. the probability that an occurring diagnosis was correctly predicted; the type 2 error rate), and the F1 score (i.e. the harmonic mean of precision and recall). The overall model score (total F1 score) is computed from the target-specific F1 scores as their support-weighted average. The support of a target equals its number of true positives. This gives more emphasis in the scoring function to higher prevalent target diagnoses.
(ii) **Selecting score-optimal hyperparameters.** While we fix the minimum support required for each feature, support_min_, we perform a hyperparameter search by applying BO to find optimal values for the remaining hyperparameters, the smoothing parameter *α* (a regularizer in the MNB estimator) and the threshold minIDP_min_ for our mutual information measure. Fivefold cross-validation was applied (a) to mitigate overfitting and (b) to reduce the noise in the total F1 score. During cross-validation, a target is selected for scoring if it occurs with at least 200 patients in every fold. A change of folds by chance in repeated model computations, leads to slightly fluctuating numbers of selected targets. To avoid a scoring bias due to changing numbers of scored target diagnoses, we fix the targets to be included in the model scoring to the top-supported 1000 targets. We refer to features in the final model, after the hyperparameters have been fixed, as the optimal feature set, $F^{*}$. See electronic supplementary material, text 3 and associated supporting figures for a more detailed algorithmic breakdown.

### 5.4. The generalized disease network

From the optimal feature set $F^{*}$ and the optimal hyperparameters from §5.3, a final predictive model was fit to the whole dataset, the *all-data model*. See electronic supplementary material, text 4. The non-trivial information contained in the correlation structure of the model coefficients of the all-data model was then used to produce a network of diagnosis features as follows.

There, nodes correspond to diagnosis features and links indicate that the mutual information of their coefficient vectors is statistically significant. The corresponding null model takes transitive relations between features into account, i.e. links are included if they (a) tend to predict similar target diagnoses and (b) are not explained through other associations between features.

Trivial associations here mean, that they can be explained transitively through other associations in the network. For example, if the three features A, B and C have identical pairwise association strengths, then each of their pairwise associations can be trivially (transitively) explained through the remaining other two associations.

The unfiltered disease network **Φ** quantifies the similarity of two features *f*_1_ and *f*_2_ in terms of the diagnoses they predict. Entries in the *N_F_* ×  *N_F_* weighted adjacency matrix Φ(*f*_1_, *f*_2_) are given by the Gaussian approximation to mutual information $\Phi (f_{1},f_{2})\;\;:=\;-1/2\;\log[1-\rho^{2}(f_{1},f_{2})],$ computed from the Pearson correlation coefficient *ρ*(*f*_1_, *f*_2_) of features *f*_1_ and *f*_2_, computed from the row vectors *C*(*f*_1_, ·) and *C*(*f*_2_, ·), respectively.

To adjust for multiple testing and class imbalance, we filter **Φ** using a network backboning approach based on Gemmetto *et al*. [69] where we proceed as follows: (i) Initialize the weights matrix **W** with elements *w*_i,j_ from **Φ**, omitting self-loops, i.e.

$$w_{i,j}\;\;:=\;\{\begin{array}{cc} \Phi (i,j) & \mathrm{if}\;i\neq j\\ 0\; & \mathrm{if}\;i=j \end{array}\;\;\forall \;i,j.$$

(ii) Discretize the weights matrix **W** by binning each entry into one of 30 bins in the range [min*_i,j_ w_i,j_*, max*_i,j_ w_i,j_*]. Note that by construction, **Φ** is non-negative and symmetric. The number of bins is chosen such to balance the performance of subsequent filtering procedure (decreases with increasing bin count) and the resolution of the discretization. (iii) Compute the irreducible maximum entropy backbone from the corresponding weighted configuration model (WCM) of the network as follows. The WCM is the maximum entropy ensemble of weighted networks that results from fixing the node strengths. The strength of node *i* is $s_{i}\;:=\underset{j}{\sum }w_{i,j}$.

(a) Vector $y=(y_{1},\;\ldots ,\;y_{N_{f}})$ (*N_f_* is the number of features) is initialized with random numbers in [0, 1). (b) We compute

$$y^{⋆}:=\mathrm{argmi}n_{y}{\left(\underset{\begin{array}{c} j\\ \left(\;j\neq i\right) \end{array}}{\sum }\frac{y_{i}y_{j}}{1-y_{i}y_{j}}-s_{i}\right)}_{i=1,\ldots ,N_{f}},$$

using an extended Levenberg–Marquardt minimizer [70]. At the minimum, the term in parentheses (i.e. the residual) is very close to zero. (c) Compute the probability (under the null model) of generating a link between nodes *i* and *j* with a weight equal to or greater than the observed weight as $\gamma_{i,j}\;:={\left(y_{i}^{⋆}y_{j}^{⋆}\right)}^{w_{i,j}}$. This corresponds to the ‘local filter’ from Gemmetto *et al*. [69, eqn 15] (but with $p_{i,j}=y_{i}^{⋆}y_{j}^{⋆}$for the WCM instead of the enhanced configuration model where also the degree sequence is fixed). (d) Filter the network **Φ** by keeping only edges (*i*, *j*) with *γ_i,j_* <  0.05.

(iv) Apply Louvain modularity detection [71] on the backbone and present its giant component with coloured clusters as the resultant generalized disease network (figure 2).

### 5.5. Quantifying synergistic interactions

The interaction strength of sub-features that make up a feature is quantified by the lift, *L*(*f*,*t*). For a feature *f* and a target *t*, the lift is given by the difference of the corresponding model coefficient and its expected value

$$L(\;f,\;t)\;:=C(\;f,\;t)-\bar{C}(\;f,\;t).$$

Let F be the ordered optimal feature set and F(*f*) be the set of diagnoses of feature index *f*. The expected coefficient is given by F$\bar{C}(\;f,\;t)\;:=\sum_{g\in G}C^{\left|F\right|}(g,\;t)\;$, where $C^{|F(g)|}(g,t)$ is the coefficient from the model of (lower) order |F(g)|. G is a partition of the set of diagnoses of feature *f*, constructed as follows. Given *f*, *G* is constructed by starting at feature index *f* and walking down F and collecting all feature indices *g* into *G* until $U_{g}\in G^{F(g)}=F(f)$. The ordering of F guarantees ⋂_*g*_ ∈ *G*${\;}^{F}$^(*g*)^ = ∅.

The coefficient $\bar{C}(\;f,t)$ can be thought of as the expected coefficient based on the linearity of the model under the assumption that *f* itself would not have been included in the optimal feature set. For non-interacting diagnoses $\bar{C}(\;f,t)=C(\;f,t)$. Positive lifts indicate synergy; negative lifts redundancy.

### 5.6. Matched cohort comparison

The two largest clusters of obesity (E66), in terms of number of features, are selected for comparison with non-obese controls. We select patients from the study population who are positive in any of the cluster's features during the feature period into the corresponding cluster cohort. The controls are all patients who are non-obese during the feature period. All patients were uniquely assignable to one of the three cohorts. To compare these cohorts while adjusting for the factors age, sex and place of residence, we take the smaller of these groups, in terms of patient number, and randomly match each patient therein to three individuals from the larger group and to 10 controls, with same sex, region and a maximum age difference of 2 years. A patient could only be used once for matching. If there are not sufficient matchable patients in the larger group, then the patient is excluded. This affected 41 patients (4.4%).

We compared the cohorts in terms of their prevalence of metabolic disorders and selected further disorders during the feature period. We also analysed the incidence of new metabolic and cardiovascular diseases in the 3-year target period. With regard to mortality, we compared the cohorts over a 9-year follow-up period (2006–2014).

### 5.7. Software and source code

To perform frequent itemset mining, we used Borgelt's Apriori for Linux, revision 6.27 1 August 2017 [66].

Major parts of the analysis are bespoke using the programming languages Python 3.7.5, Ruby 2.6.5 and Rust 2018. The software is modular and uses the package management systems Poetry, Rubygems and Cargo. The used package versions are documented in the respective package manager files in the source code repository of this work. The source code of this study is available at https://github.com/mstrauss/rsif20201040.

To compute the network layout and the Louvain modularity, we used Gephi 0.9.3-SNAPSHOT 201810261216/Force Atlas 2.

## Data accessibility

The data that support the findings of this study are available from GÖG but restrictions apply to the availability of these data, which were used under license for the current study, and so are not publicly available. Data are however available from GÖG (www.goeg.at) upon reasonable request and with permission.

At the time of publication, the contact for data requests is Michael Gyimesi, Michael.Gyimesi@goeg.at. At the time of publication, the contact for data requests is Michael Gyimesi, Michael.Gyimesi@goeg.at.
